# Crystalline Mesoporous F-Doped Tin Dioxide Nanomaterial Successfully Prepared via a One Pot Synthesis at Room Temperature and Ambient Pressure

**DOI:** 10.3390/nano13192731

**Published:** 2023-10-09

**Authors:** Tariq Aqeel, Heather F. Greer

**Affiliations:** 1 Department of Science, College of Basic Education, The Public Authority of Applied Education and Training (PAAET), P.O. Box 23167, Safat 13092, Kuwait; 2 Yusuf Hamied Department of Chemistry, University of Cambridge, Cambridge CB2 1EW, UK; hfg24@cam.ac.uk

**Keywords:** fluoride doping, mesoporous tin dioxide, high conductivity, opto-electrical properties

## Abstract

We report the successful one pot synthesis of crystalline mesoporous tin dioxide powder doped with fluoride at ambient pressure and temperature. This material possesses a high surface area, narrow pore size distribution, small average crystallite sizes, and good opto-electrical properties. The existence of fluorine increased the opto-electronic activity of tin dioxide by 20 times, and conductivity by 100 times compared with pristine tin dioxide prepared via the same method. The conductivity of SnO_2_ in air at 25 °C is 5 × 10^−5^ S/m, whereas that of F–SnO_2_ is 4.8 × 10^−3^ S/m. The structures of these materials were characterized with powder X-ray diffraction, N_2_ sorption analysis, transmission electron microscopy, scanning electron microscopy, energy dispersive X-ray spectroscopy, X-ray photoelectron spectroscopy, and UV-visible spectroscopy. Fluorine occupies the framework of tin dioxide by replacing some of the oxygen atoms. The structure, conductance, and optical properties of these materials are discussed in this paper.

## 1. Introduction

Crystalline tin dioxide is an n-type semiconductor material possessing many interesting physical and chemical properties, for example, a wide band gap of 3.6 eV [1,2] and an active surface employed for gas sensing [3,4,5] and in opto-electronic reactions [6,7,8]. It is widely used due to its relatively cheap cost, wide range of conductance, operational stability, and fast charge transfer [9]. Changing the surface chemistry or the morphology will affect the band gap and, as a result, the conductance and optical properties of the material. The band gap of a semiconductor can be affected by many factors, such as doping with other elements [7,10,11], creating more structural defects [11,12] or changing the crystallite sizes [13,14]. In addition, increasing the surface area of this material by synthesizing a porous structure will enhance the diffusion of gasses and fluids [15] that would interact with these surfaces during chemo sensing or opto-electronic catalysis. It was found that creating mesoporous tin dioxide (SnO_2_) increases the surface area from 10 m^2^g^−1^, as a non-porous material [16], to about 200 m^2^g^−1^ [17]. It was found that controlling the surface area is highly dependent on the selected heat treatments employed during the calcination step of the synthesis [18]. As a result, the crystallite sizes of SnO_2_ are also affected by this heat treatment [10,19]. Creating a high surface area and decreasing the crystallite sizes is known to improve surface sensitivity [20]. Moreover, producing porous SnO_2_ with chemically connected crystallites will improve the charge transfer in the material, which will improve the conductivity and the opto-electronic activity of the material [10,21].

On the other hand, placing foreign elements in the framework may improve the electrical conductance by increasing surface charges [22,23] and decreasing the band gap by creating an additional defect band under the conduction band or above the valence band [10,20,23]. Each foreign element (for example the fluoride ion, F^−^) will introduce specific properties to SnO_2_ [23]. The fluoride ion, for example, would replace some of the oxygen atoms in the framework [24]. Replacing oxygen atoms with fluoride ions will strengthen the framework. This is because fluorine has higher electronegativity than oxygen [25], which means the bonds formed with F^−^ are shorter than those formed by O^2−^ [10]. This improves the overall connectivity of the structure and enhances charge transfer [10]. Moreover, F^−^ is considered to be a single electron donor, which will add more electrons to the conducting bands [24]. This will enhance the conductivity of SnO_2_, by making more structural defects such as oxygen vacancies [22]. This would introduce an additional “defect band” under the main conduction band [26]. This intermediate defect band decreases the main gap, allowing more valance electrons to hop and conduct electricity at lower energies [13,14,16]. Decreasing the band gap is another key factor affecting the implementation of semiconductor materials. As a result, these materials will require less activation energy to react or conduct electricity. The original band gap of tin dioxide is 3.6 eV [1,2] and it requires a high energy photon of ultraviolet light (at or lower than 344 nm wavelength) to free electrons from the valence band to reach the conductance band. However, decreasing the band gap, for example to ~3 eV, will make the material optically excited in the violet visible wave range (at 413 nm). This means that the material could be employed in the visible solar spectra as well as in the UV range. This is important for the usage of this material in photovoltaic cell applications, in visible light detectors/instruments, and in visible optical/catalytic reactions.

We have improved the original precipitating synthesis method of SnO_2_ powders first introduced by Severin et.al. [27]. Severin’s method produced a porous structure of SnO_2_ that collapsed when calcined above 350 °C. Our modified method, which is presented in this work, produces mesoporous SnO_2_ crystalline material that can undergo multiple heat treatments up to 500 °C and is highly reproducible [17]. Therefore, we successfully employed our modified method in this project to synthesize crystalline powders of mesoporous F–SnO_2_ possessing a high surface area and a small crystallite size. This material’s characterization and the opto-electronic properties are studied and discussed in this paper.

## 2. Materials and Methods

Mesoporous F–SnO_2_ was prepared by slowly stirring hexadecylamine (0.27 g) in isopropanol (^i^PrOH (24.4 mL)) until completely dissolved. Next, tin (IV) isopropoxide (Sn(O^i^Pr)_4_ (1.9 g)) was added to the solution at room temperature (25 °C), followed by ammonium fluoride (H_4_NF (0.05 g)). The reactants were stirred slowly under water-saturated air (approximately 80% humidity) at atmospheric pressure for 3 days. The obtained product was filtered and washed with water and ethanol. The product was then transferred to a Soxhlet extractor with ethanol overnight (approximately 16 h) to remove the surfactant, and was subsequently collected via filtration. The product was then calcined at 300 °C in air for 0.5 h then to 400 °C for 15 min (heating rate of 2 °C min^−1^) and subsequently labeled F–SnO_2_. A similar procedure was followed to produce mesoporous tin dioxide, but without the addition of H_4_NF, labeled SnO_2_. All chemicals used were purchased from Alfa Aesar, were of analytical grade and were used without further purification.

### Characterization

Bruker AXS D8 ADVANCE diffractometer (Bruker, Billerica, MA, USA) (Cu Kα radiation *λ* = 1.5418 Å) was employed to carry out powder X-ray diffraction (XRD) measurements. Parameters were set at 40 kV, 40 mA, 0.1 mm low-angle front slit window size, 1.0 mm for wide-angle scans, 0.5–1.0 mm gap between the sample and the deflection plate, and for low-angle scans, 0.5 steps∙s^−1^ in continuous coupled two-theta scan/theta scan mode. DIFFRAC^plus^ software (version V3.1) was used for XRD analysis. A Micrometrics Tristar analyzer was used for nitrogen gas sorption analyses. A JEOL JEM-3010 microscope (JEOL, Akishima, Japan) operating at 250 kV was used to obtain transmission electron microscopy (TEM) images. TEM images were recorded using a Gatan 794 CCD camera and analyzed with ImageJ software (version V1.53). Specimens were suspended in acetone and one drop placed on a 300 mesh Cu grid containing a holey carbon film. Scanning electron microscopy (SEM) and energy dispersive X-ray spectroscopy (EDX) spectra were obtained using a TESCAN MIRA3 FEG-SEM equipped with an Oxford Instruments Aztec Energy X-maxN 80 EDS detector (Oxford Instruments, Abingdon, UK). The specimens were placed on adhesive carbon mounted on an Al pin stub. A Thermo ESCALAB 250Xi spectrometer (Thermo, Waltham, MA, USA) with a monochromator and Al-Kα radiation source (1486.6 eV) was employed for X-ray photoelectron spectra (XPS). The XPS spectra was recorded and processed using an Avantage data system at set parameters of 20 eV pass energy, 10^−9^ Torr analysis chamber pressure, 100 ms dwell time, and a 0.1 eV step size. The binding energy values were determined using C 1s line (284.6 eV) of adventitious carbon. A flood gun was used to neutralize the charge buildup on the surface of the insulating layer in the standard charge compensation mode. To perform UV tests, the samples were pressed into 10 mm diameter pellets. A Cary 5000 UV-Vis-NIR spectrophotometer (Agilent, Santa Clara, CA, USA) (version 1.12) was used to perform the UV-Vis spectroscopy at a scan rate of 600.0 nm min^−1^, Abs mode 200–800 nm, data interval of 1.0 nm, full slit height, baseline correction on, double-beam mode, and signal-to-noise mode off. The UV excitation/conductivity test was performed employing a UniEquip UV source of a Hg tube lamp (365 nm, 50–60 Hz, 24 W, and 230 V). The current was measured using an Agilent B2901A source/measure unit (Agilent, Santa Clara, CA, USA) and two banana probes placed 4 mm apart at room temperature.

## 3. Results and Discussion

### 3.1. XRD

**Figure 1:**
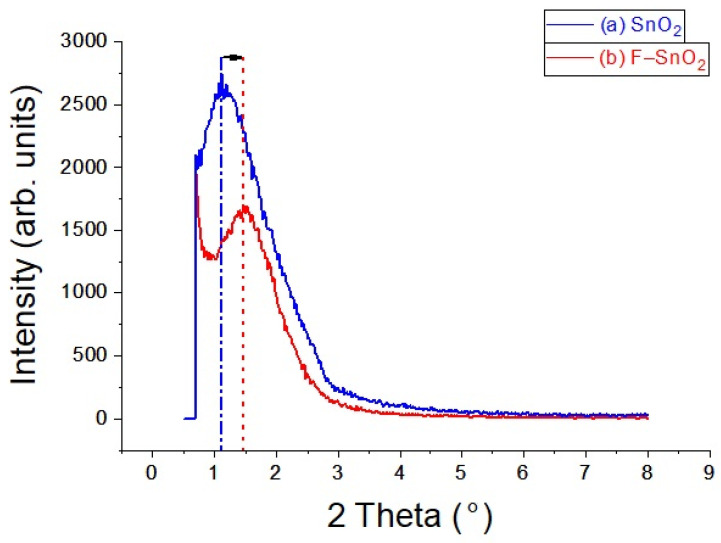
Low-angle XRD patterns of (**a**) SnO_2_ and (**b**) F–SnO_2_.

Low angle XRD patterns presented in Figure 1 reveal that both samples, SnO_2_ and F–SnO_2_, have mesoporous structures, from the presence of the main diffraction peak at (100). This peak shifted towards a larger angle when SnO_2_ was doped with fluorine, which indicates a slight decrease in the unit cell (a_0_) as a result of replacing some of the O atoms with F. The reduction in *d*-spacing (presented in Bragg’s law Equation (1)) is indicated by the dotted lines and the arrow in Figure 1.

d_100_ = n λ/2 sinθ (1)

d_100_ = a_0_ sin60 (2)

where n is an integer, λ is the incident X-ray wavelength, and θ is the diffraction angle. The a_0_ in a porous structure represents a diffraction plane of straight distance between two adjacent pore centers that are separated by a wall thickness of material. The calculated a_0_ for SnO_2_ was 85.2 Å, whereas it was 68.5 Å for F–SnO_2_ Å. This decrease in a_0_ was the result of two factors: having a narrower pore size and incorporating fluorine atoms in the framework, which was more electronegative than oxygen [28,29]. This will shrink the crystallite sizes and the thickness of the wall between the pores. Notice that both samples underwent similar synthesis procedures and heat treatments. Moreover, the shapes of the main (100) diffraction peak for both materials SnO_2_ and F–SnO_2_ were similar, indicating that F atoms were well incorporated in the framework and did not upset the overall pore ordering in the structure. No other peaks were observed in this scan, indicating that there was no long-range ordering of the pores. This also indicates that most of the pores did not form tube-like shapes.

**Figure 2:**
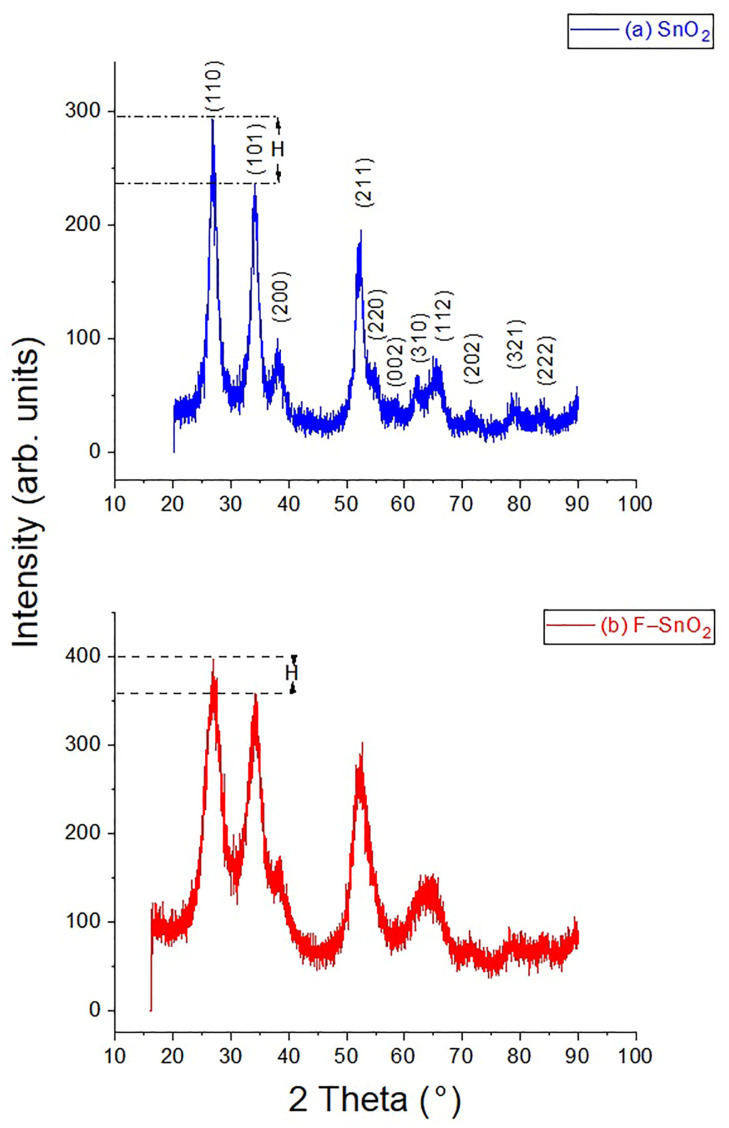
Wide angle XRD patterns of (**a**) SnO_2_ and (**b**) F–SnO_2_.

The wide-angle XRD patterns presented in Figure 2 show that both samples have a crystalline nature and that pure SnO_2_ was synthesized [30]. The diffraction peaks for both samples were indexed to the Cassiterite (tetragonal) structure of SnO_2_ [31,32]. No additional diffraction peaks appeared, indicating that the F species did not cluster or form a secondary phase outside the framework. This also confirms that the substitution of F ions did not affect the crystallinity of the material, [19,30] and that F ions were inserted into the framework [10]. Moreover, F^–^ favors the (101) plane [10,24], which is why the intensity of this plane was relatively higher in relation to the (110) plane for F–SnO_2_ in comparison with these planes of SnO_2_. That is why the height difference (H) was relatively smaller in F–SnO_2_ than in SnO_2_, as presented in Figure 2a,b. This also confirms that F^−^ is readily integrated in the framework of SnO_2_. Moreover, the (110) diffraction peak was used to calculate the average crystallite sizes for both samples by employing the Scherrer equation. The calculated average crystallite sizes were 4.1 and 3.4 nm for SnO_2_ and F–SnO_2_, respectively. The difference in the crystallite sizes agrees with and confirms the variation in the value of the unit cell a_0_ calculated in the low-angle XRD scan. These crystallite sizes confirm that SnO_2_ and F–SnO_2_ are both nanomaterials.

### 3.2. N_2_ Sorption Analysis

**Figure 3:**
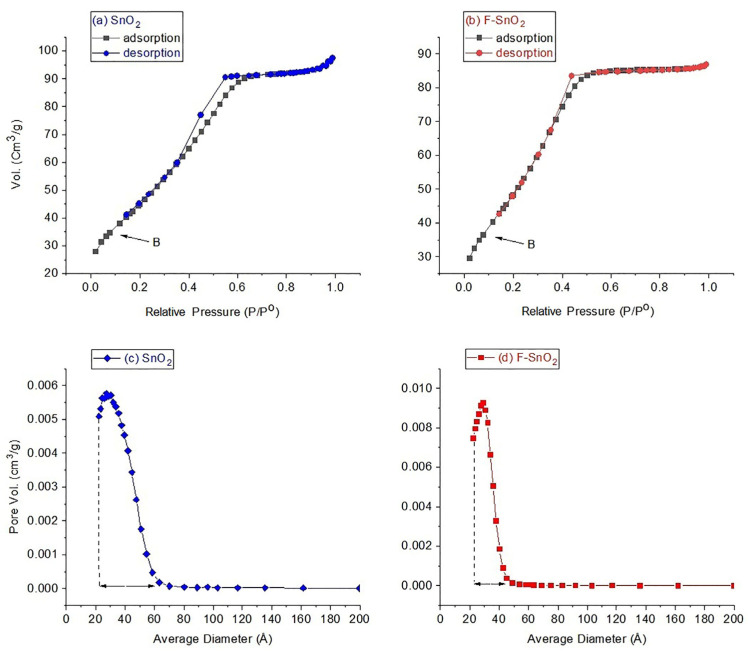
N_2_ sorption isotherms of (**a**) SnO_2_, (**b**) F–SnO_2_, and the corresponding pore size distributions of (**c**) SnO_2_ and (**d**) F–SnO_2_.

N_2_ adsorption/desorption analysis was performed on both materials, SnO_2_ and F–SnO_2_, with the resultant isotherm curves presented in Figure 3a,b. Both samples produced a type IVa isotherm [33], which was assigned to mesoporous materials. There were distinctive features of these isotherms, that the start of inflection point B (at 0.1 relative pressure) in these materials was not sharp, which is indicative that the mono layer was not completely covered by N_2_. Then, the coverage of N_2_ was extended for the mono and multi-layer at 0.2–0.6 relative pressure. After that, N_2_ capillary condensation occurred inside the pores before reaching the saturation pressure (p^0^), which was represented by the plateau between 0.6 and 0.9 relative pressure. Finally, a hysteresis curve (typical of IVa isotherms) occurred in the desorption cycle, which indicated the existence of some pores that exceeded the critical size of 4 nm in both materials. This hysteresis occurred when there was a slower rate of desorption than adsorption of N_2_. It is worth mentioning that the hysteresis curve was larger in the SnO_2_ sample than in F–SnO_2_, indicative that there were more large pores (larger than 4 nm) in SnO_2_ than in F–SnO_2_. This was also confirmed by the pore size distribution (Figure 3c,d), showing that sample SnO_2_ had a wider pore size distribution, falling in the range 2–6 nm. In contrast, the pore distribution in F–SnO_2_ was narrower, falling between 2 and 4 nm, as presented in Figure 3d.

Moreover, the BET surface areas obtained from SnO_2_ and F–SnO_2_ samples were 164 and 180 m^2^g^−1^, and the average pore size was 36.2 and 29.8 Å, respectively, presented in Figure 3c,d. Both N_2_ sorption results, the isotherm type and the pore size diameter, confirmed that SnO_2_ and F–SnO_2_ samples were classified as mesoporous materials according to IUPAC [33].

### 3.3. TEM

**Figure 4:**
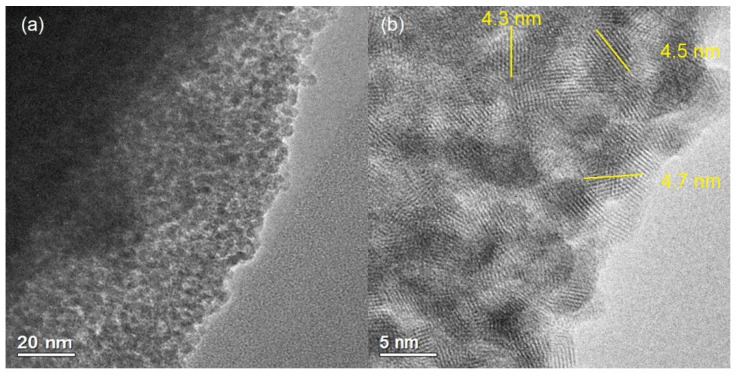
(**a**) TEM and (**b**) HRTEM images of SnO_2_.

TEM images were acquired of SnO_2_ and F–SnO_2_ to give an insight into the crystallinity, pore location, and distribution in these materials. Figure 4a reveals that the pores were well distributed in SnO_2_ but were not uniform in size or shape, and SnO_2_ material surrounded these pores. Figure 4b confirmed that SnO_2_ is a crystalline material, which consists of semi-spherical crystallites that are fused together around the pores. Moreover, these crystallites vary in size and it is hard to determine the exact sizes of these crystallites using TEM images. Despite that, we managed to measure a few crystallite sizes that averaged to 4.5 nm, as indicated by yellow lines in Figure 4b. This size is similar to that calculated from wide-angle XRD.

**Figure 5:**
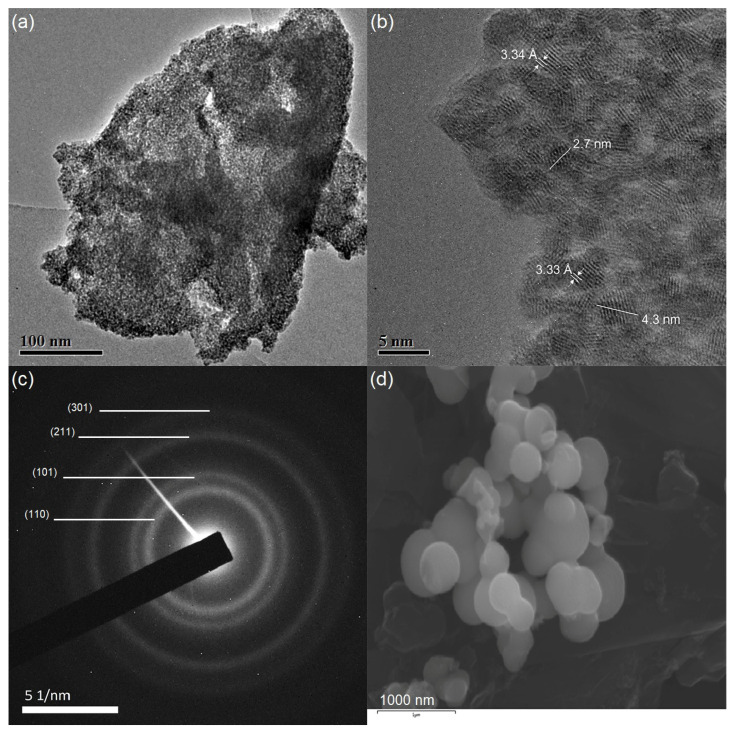
(**a**,**b**) TEM/HRTEM images of F–SnO_2_, (**c**) selected area electron diffraction pattern acquired from the particle in (**a**,**b**), and (**d**) SEM image of F–SnO_2_.

Figure 5 represents TEM/HRTEM images acquired from F–SnO_2_. Figure 5a confirmed that the material contained pores, which spread across entire particles. Figure 5b is an HRTEM image of the particle in (a), showing crystalline fringes of 3.34 Å that can be assigned to the (110) plane of SnO_2._ In addition, the sample contained semi-spherical shaped crystallites that were fused together in a framework around the pores. The sizes of these crystals fell between 2.7 and 4.3 nm, as presented in Figure 5b, which agreed with the wide angle XRD calculation. Moreover, HRTEM images did not reveal any aggregates of F species or appear substantially different from SnO_2_, which further confirms that F^−^ was substituted into the framework. Figure 5c shows the electron diffraction pattern acquired from the particle in Figure 5a, indicating the polycrystalline structure of F–SnO_2_ with the diffraction rings indexed to tetragonal SnO_2_ [34]. The SEM image in Figure 5d confirmed that the semi-spherical shape of the F–SnO_2_ crystals were uniform and this shape was maintained to the micro level. This also confirmed that these micro grains were chemically fused together. This stacked growth promotes charge transfer.

### 3.4. EDX

**Figure 6:**
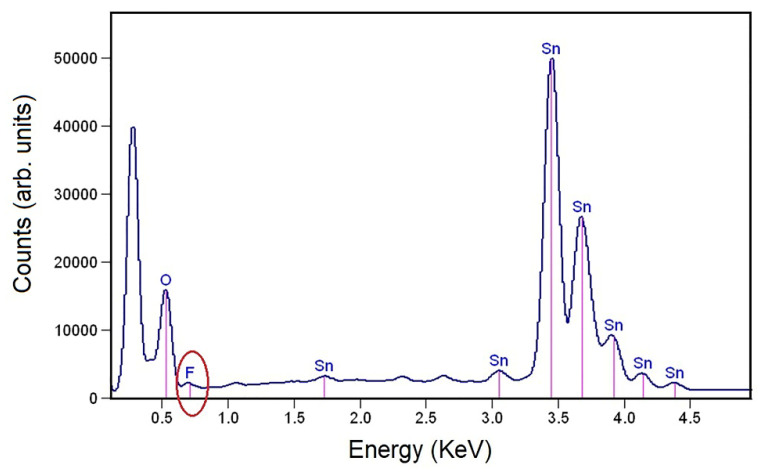
EDX spectrum of F–SnO_2_ sample.

The EDX spectrum shows the K and L peaks of all the elements present in the F–SnO_2_ sample, in Figure 6, which reveals that the sample composition contained Sn, O, and F atoms. This confirms that F was successfully incorporated in the F–SnO_2_ sample and it was part of its composition. The circled peak in Figure 6 clearly shows the K_α_ peak of F at 0.67 keV, which was adjacent to the K_α_ O peak and had almost been masked by it. The bonds and the chemical composition of these elements would be determined using XPS.

### 3.5. XPS

**Figure 7:**
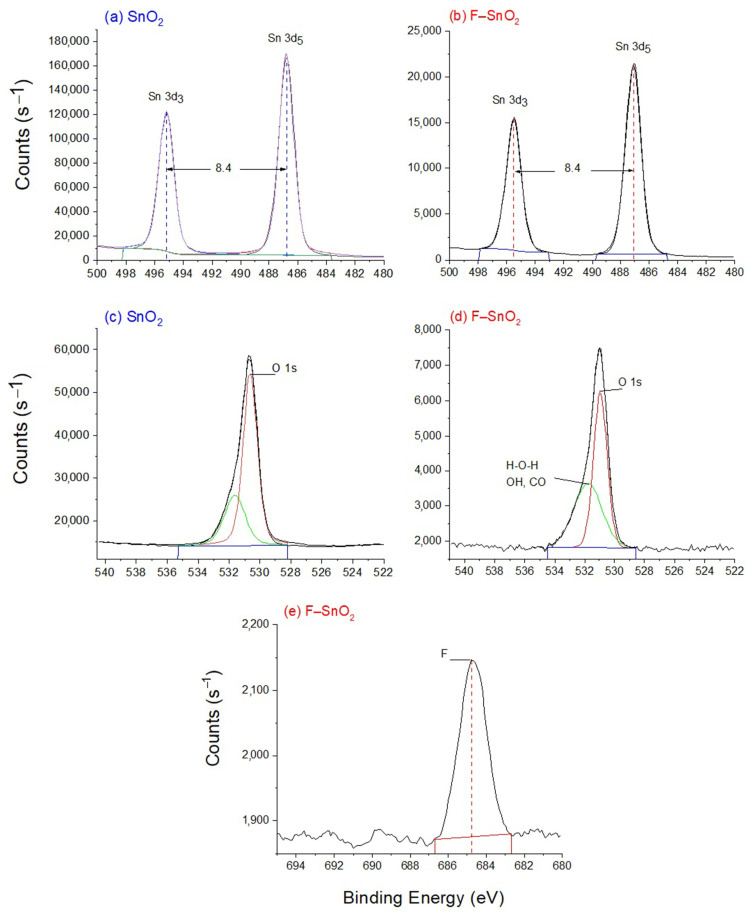
XPS spectra for SnO_2_ and F–SnO_2_ (**a**,**b**) Sn 3d, (**c**,**d**) O 1s, and (**e**) F 1s regions.

XPS analyses (Figure 7) were performed for both materials to investigate the surface and partial subsurface compositions and their oxidation states. XPS results in Figure 7a,b for both materials, SnO_2_ and F–SnO_2_, showed spin-orbit doublet peaks at 486.6 (Sn 3d_5/2_), 495.0 (Sn 3d_3/2_), and 487.1, 495.5 eV, respectively, plus 3d orbital splitting of 8.4 eV. This corresponded to the Sn^4+^ oxidation state of SnO_2_ [32,35]. The slight increment of 0.5 eV in the peaks of Sn 3d of the F–SnO_2_ sample could be attributed to the effect of the presence of the more electronegative F ions in the sample [10,36]. The XPS peak of O 1s spectra at 530.6, and 530.0 eV for SnO_2_ and F–SnO_2_, respectively, represented the lattice O^2−^ species of SnO_2_ for both samples, Figure 7c,d [35,37]. The second O 1s XPS peak for both samples, at 531.6 and 531.7 eV, respectively, represented adsorbed surface oxygens that existed in the atmosphere, such as OH, H_2_O [37], and CO_2_ [34]. This did not exclude the presence of oxygen vacancies which had the same XPS binding energies around these values, 531.7 eV [38]. This was also confirmed by the atomic ratio collected from the XPS data, O/Sn of 2.1 and 2.3 at. % for SnO_2_ and F–SnO_2_, respectively, showing extra oxygen species adsorbed at the surface for both samples, but was more pronounced for the F–SnO_2_ sample. This is because F is more electronegative than Sn, making Sn^4+^ more positively charged [32]. This will make Sn attract a more negatively charged oxygen species. Furthermore, the XPS peak at 684.6 eV in Figure 7e, whose binding energy corresponded to F 1s, which replaced some oxygen (O^2−^) in the lattice of F–SnO_2_, confirms that F^−^ became a single electron donor [10,24]. In addition, the F^−^ wt./wt. percentage calculated from XPS data was 1.1% F/SnO_2_ (1.4% F/Sn), which was less than the intended percentage calculated theoretically during the synthesis procedure, which was 3%. This decrease may occur during the washing of the as-synthesized sample or during the Soxhlet extraction steps, both of which were performed before the calcination step. During calcination, more condensation occurred in the framework that strengthened the bonds between the atoms in the structure. Therefore, we think that the lost F species were superficial and they did not form bonds deep in the structural framework.

### 3.6. UV-Visible Spectroscopy

**Figure 8:**
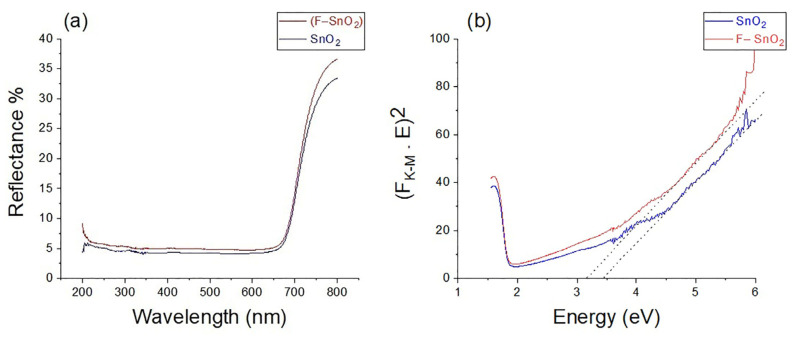
(**a**) Reflectance % and (**b**) the Tuck plot for SnO_2_ and F–SnO_2_, respectively.

The diffuse reflectance (R) Figure 8a was performed for both samples to be converted into the Kubelka–Munk function (F_K–M_) to produce the Tauck plot. The reflection of both materials was less than 7% in the UV until the red-light range, at which both reflections increased above 30% (Figure 8a). This indicates that both materials had high transmission values in these regions [28]. The constructed Tauck plot in Figure 8b was employed to determine the band gap of these materials, basically plotting (F_K–M_ × E)^2^ against the energy (E) at which the intercept of the tangent gives the direct optical band gap of the material [39,40,41].

The following equation explains the F_K–M_:

F_K–M_ (R) = (1 − R)^2^/2R (3)

E = *hν* (4)

(*αhν*)^2^ = A (*hv* − E_g_) (5)

where *α* is the absorption coefficient, *h* is Plank’s constant, *v* is light frequency, A is constant, and Eg is the direct band gap energy [38]. F_K–M_ = *α/s*, where *s* is the scattering coefficient. The band gaps for SnO_2_ and F–SnO_2_ were 3.40 and 3.16 eV, respectively, as shown by the tangent dotted lines in the Tauck plot shown in Figure 8b. The lower band gap of both materials could be the result of many factors. First, having long chemically connected structures (chain-like) reduces the band gap [42]. The second factor is because of strains caused by having SnO_2_ thin walls, which narrow the gap distance between conduction and valence bands [43]. Third is the probability of having an intermediate SnO_x_ band [44], and the existence of an intermediate defect band of oxygen vacancies that appears above the valence band of SnO_2_ [12,13,45]. This also confirms that doping SnO_2_ with F^−^ decreases the bandgap, most likely by creating a defect band below the conductance band [44,46,47]. On the other hand, these bandgap values suggest that SnO_2_ and F–SnO_2_ would be optically excited by UV-A light at and below 365 and 392 nm, respectively. Furthermore, this 392 nm wavelength calculated for F–SnO_2_ was very close to the visible violet border wavelength of 400 nm, which was the border of visible light.

### 3.7. Conductivity and Opti-Electronic Tests

The conductivity of both materials, SnO_2_ and F–SnO_2_, was measured in air at 25 °C with the conductance being 5 × 10^−5^ and 4.8 × 10^−3^ S/m, respectively. This confirms that adding 1% of F^−^ to SnO_2_ increased the conductivity by 100 times. By donating more free charges to the framework, and strengthening the framework connectivity (as discussed in Section 3.3), this facilitated charge transfer.

**Figure 9:**
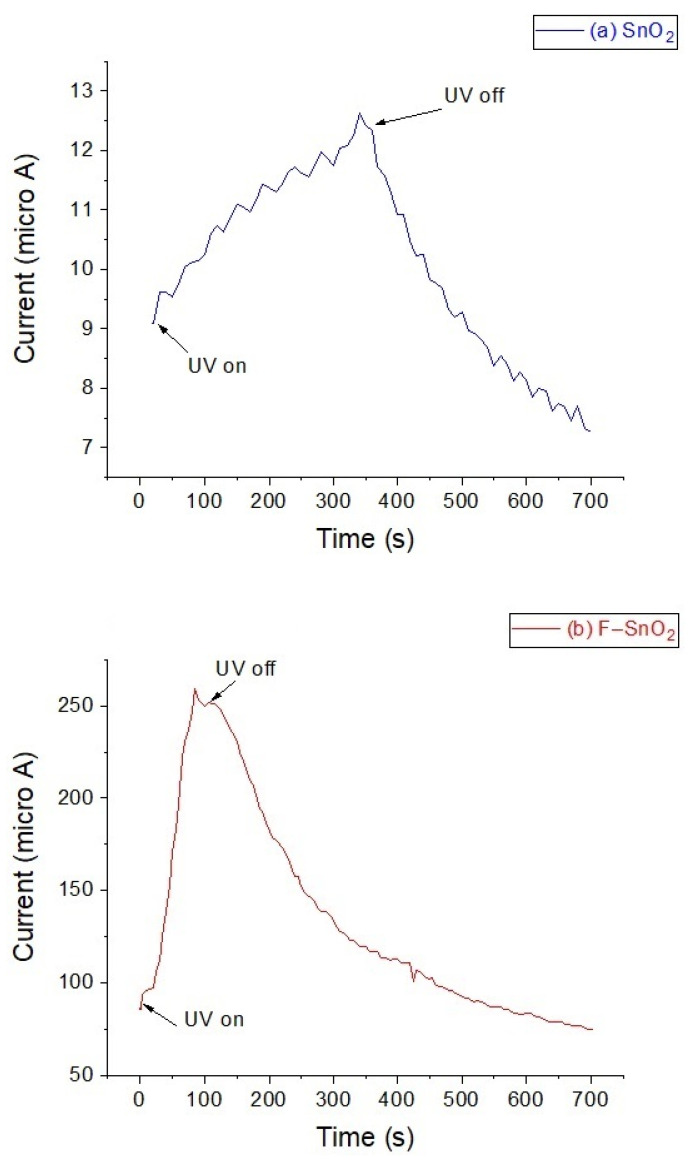
Current produced per second by exposing the samples to UV radiation: (**a**) SnO_2_ and (**b**) F–SnO_2_.

To study the optoelectronic excitation effect of the materials, both samples were subjected to UV light for a few minutes, while the current produced was recorded simultaneously, as presented in Figure 9. This current was a measure of the excited electrons (e^−^) coming from the valence band hopping to the conduction band for the semiconductor materials [38]. Initially, SnO_2_ was exposed to UV light for 5 min, at which point the current increased to a maximum of 12.6 μA. After that, the UV source was switched off and the recorded current continued to decline, as shown in Figure 9a. F–SnO_2_ was then subjected to the same source for 2 min, and when the UV light was on the current increased sharply from 70 to 259 μA. Next, this source switched off and the current started to decline gradually to the end of the test (Figure 9b). This produced a current that was about 20 times larger than that of SnO_2_. This test confirmed two points, firstly that both samples can be optically excited under wavelengths at/or below 365 for SnO_2_ and 392 nm for F–SnO_2_. Secondly, F–SnO_2_ is more optically active than SnO_2_ at similar experimental conditions; this could be the result of having a smaller band gap. This smaller band gap allowed more charges to be excited at this wavelength (365 nm) than in pristine SnO_2_. This could be the result of having an electron donor fluoride ion in the framework, more charges available, and faster charge transfer because of a more compacted structure.

## 4. Conclusions

We successfully synthesized a crystalline mesoporous tin dioxide nanomaterial doped with fluoride in one pot at an ambient pressure and temperature (using our modified method). The crystallites of this material were chemically connected in a continuous framework. F–SnO_2_ possessed high electrical conductivity in air 4.8 × 10^−3^ S/m, which was 100 times larger than pristine SnO_2_. F–SnO_2_ was approximately 20 times more optically active than pristine SnO_2_ under a UV source. As a result of having F^−^ in the framework, this made the structure more compact and connected. In addition, F^−^ is an electron donor ion. Moreover, F–SnO_2_ has a high BET surface area of 180 m^2^g^−1^, an average crystallite size of 3.4 nm, and a narrow pore size distribution of 2–4 nm, which promotes this material to be readily employed in optoelectronic cells and instruments.

## Data Availability

Not applicable.
